# Energy Structure and Luminescence of CeF_3_ Crystals

**DOI:** 10.3390/ma14154243

**Published:** 2021-07-29

**Authors:** Orest Kochan, Yaroslav Chornodolskyy, Jarosław Selech, Vladyslav Karnaushenko, Кrzysztof Przystupa, Aleksei Kotlov, Taras Demkiv, Vitaliy Vistovskyy, Hryhoriy Stryhanyuk, Piotr Rodnyi, Alexander Gektin, Anatoliy Voloshinovskii

**Affiliations:** 1 School of Computer Science, Hubei University of Technology, Wuhan 430068, China; orest.v.kochan@lpnu.ua; 2 Department of Measuring Information Technologies, Lviv Polytechnic National University, Bandery Str. 12, 79013 Lviv, Ukraine; 3 General Physics Department, Ivan Franko National University of Lviv, 8 Kyryla i Mefodiya, 79005 Lviv, Ukraine; yaroslav.chprnodolskyy@lnu.edu.ua (Y.C.); vladyslav.karnaushenko@lnu.edu.ua (V.K.); taras.demkiv@lnu.edu.ua (T.D.); vitaliy.vistovskyy@lnu.edu.ua (V.V.); anatoliy.voloshinovskii@lnu.edu.ua (A.V.); 4 Institute of Machines and Motor Vehicles, Poznan University of Technology, 60-965 Poznan, Poland; jaroslaw.selech@put.poznan.pl; 5 Department of Automation, Lublin University of Technology, Nadbystrzycka Str. 36, 20-618 Lublin, Poland; 6 Deutsches Elektronen-Synchrotron DESY, 22607 Hamburg, Germany; aleksei.kotlov@desy.de; 7 Helmholtz Centre for Environment Research, 15 Permoserstr, 04318 Leipzig, Germany; gregory.stryhanyuk@ufz.de; 8 Physics Department, Peter the Great St. Petersburg Polytechnic University, 29, Polytekhnicheskaya, 195251 St. Petersburg, Russia; piotr_rodnyi@mail.ru; 9 Institute for Scintillation Materials, National Academy of Sciences of Ukraine, Nauka Ave. 60, 61001 Kharkiv, Ukraine; gektin@yahoo.com

**Keywords:** luminescence, energy structure, Frenkel self-trapped excitons, CeF_3_

## Abstract

The results of the calculation of the energy band structure and luminescent research of CeF_3_ crystals are presented. The existence of two 5d1 and 5d2 subbands of the conduction band genetically derived from 5d states of Ce^3+^ ions with different effective electron masses of 4.9 m_e_ and 0.9 m_e_, respectively, is revealed. The large electron effective mass in the 5d1 subband facilitates the localization of electronic excitations forming the 4f-5d cerium Frenkel self-trapped excitons responsible for the CeF_3_ luminescence. The structure of the excitation spectra of the exciton luminescence peaked at 290 nm, and the defect luminescence at 340 nm confirms the aforementioned calculated features of the conduction band of CeF_3_ crystals. The peculiarities of the excitation spectra of the luminescence of CaF_2_:Ce crystals dependent on the cerium concentration are considered with respect to the phase formation possibility of CeF_3_.

## 1. Introduction

The progress of science is often determined by the progress of measurements, so there is intensive research in this area [1,2,3]. The studies consider the whole measuring channel [4,5,6] and its individual components [7]. It should be noted that in modern measurements, the main contribution to the measurement uncertainty belongs to sensors [8,9]. Technologically, it is very difficult to mitigate the errors of sensors. Therefore, individual calibration [10,11] and artificial intelligence are often used [12,13] to improve the accuracy of sensors [14]. However, the problem of the instability of sensors cannot always be solved by these means because sensors are exposed to various influences in aggressive environments and acquire large errors [15,16,17]. In some cases, some methods to subside the impact of the degradation processes in sensors were developed, but this is the exception rather than the rule [18,19]. Therefore, the problem of developing new sensors and new sensor materials is very topical [20,21], especially with respect to the concept of the Internet of Things [22,23]. There is a growing need to develop better and more accurate sensors because measurement accuracy also has economic [24,25,26], environmental [27] and medical effects [28,29]. In some branches of science, this need is mentioned as key for the next decade [30,31]. One of the promising ways to solve it is the study of new materials [20,32] for sensors.

The luminescent properties of CeF_3_ crystals have been the subject of numerous scientific studies since the time this material began to be considered as a promising scintillator for detectors in the field of high energy physics [33,34,35]. In this paper, among many possible aspects, we pay attention to the features of the energy structure of the crystals and the nature of the observed luminescence. CeF_3_ crystals show that luminescence bands peaked at 290 nm and 340 nm. The luminescence bands around 290 nm (282 and 308 nm at 10 K) are attributed by many researchers to 5d-4f transitions in the Ce^3+^ ion [36], and this is a prevailing approach to the interpretation of this band. However, based on the fact that cerium ions are constitutional atoms of the crystal, it is reasonable to assume the possibility of interaction between ions and the appearance of such excitations as Frenkel excitons [37,38,39,40,41]. This approach is clearly implemented in the analysis of the transient time-resolved absorption spectra of CeBr_3_ crystals [42]. Today, this is an example of convincing evidence for the existence of the cerium 4f-5d exciton, which is observed in CeX_3_ crystals (X = F, Cl, Br, I). Quantitative energy calculations of CeX_3_ suitable for the analysis of emission transitions and the nature of transitions in the range of fundamental absorption are absent, but the available ones generally determine the regularities of the energy band formation in the CeF_3_ crystals [43,44,45,46,47,48].

It is worth noting the positive influence of qualitative energy band schemes based on the analysis of experimental spectroscopic data [35,40,49] on the understanding of the luminescent and absorption processes in CeF_3_ crystals. In particular, the concept of the formation of energy bands in CeF_3_ crystals is qualitatively presented in the work [40] and is based on the combination of energy bands formed by electron energy states in the field of cerium 4f hole and 2pF^0^ hole. Consequently, the electronic structure of CeF_3_ (as well as of other compounds with cerium as a host constituent) can be considered as a superposition of LaF_3_ states with the states of the cerium subsystem [40]. By the first-principle calculations presented in this paper, we want to focus on the peculiarities of the conduction band of crystals, which are derived from the behavior of an electron in the field of 4f cerium hole and 2pF^0^ hole of the valence band. We also try to find experimental confirmations of the features of the CeF_3_ conduction band by analyzing the dependence of the luminescence excitation spectra in CaF_2_:Ce crystals with respect to the cerium concentration. The luminescence band at 360 nm is interpreted as the luminescence of perturbed cerium ions [36] or the defect luminescence [50]. For this luminescence, we consider a possible mechanism of energy transfer from cerium excitons analyzing the decay time parameters of the exciton and defect luminescence.

## 2. Materials and Methods

Theoretical calculations of the band structure for the CeF_3_ crystal energy were carried out in the open-source software Abinit [51] using the projected augmented waves method (PAW) [52]. One of the peculiarities of lanthanide ions is that the presence of strongly localized states and the exchange–correlation energy of the states cannot be correctly described by means of local density approximation (LDA) and its gradient modification (GGA). The aforementioned functionals of exchange–correlation interaction are based on the model of a homogeneous electron gas, which cannot describe the localized 4f-states of lanthanide ions with the required accuracy. There are two approaches to solve this problem: the hybrid functional of the exchange–correlation interaction PBE0 [53] and the Hubbard corrections in the DFT+U [54] method. In the current work, the hybrid functional PBE0 is used [53]. The functional requires more computational resources but allows for the obtention of more accurate values of energy parameters due to the smaller number of approximations. This functional is represented as a sum of two components such as the PBE exchange–correlation functional [52,55] and the difference weighted by the parameter α between the Hartree-Fock exchange functional and the PBE:

(1) $$E_{xc}^{PBE0}=E_{xc}^{PBE}+\alpha \left(E_{x}^{HF}-E_{x}^{HBE}\right)$$

The calculations were performed with the cutoff energy of 40 Ha and the energy of 120 Ha for the augmented PAW using a 6 × 6 × 6 Monkhorst-Pack grid and a typical for a hexagonal cell shift (0 0 ½). The choice of the number of k points is determined by the need to describe highly localized states of lanthanides. The spin properties of the system were considered to take into account the population of 4f states. Self-consistency of calculations was achieved after 11 iterations, which confirms the correctness of the input parameters. The parameter α = 0.25 is optimal for calculations in similar systems [48].

LaF_3_:Ce, CeF_3_ and CaF_3_:Ce single crystals were grown using the Stocbarger technique in an inert atmosphere. The measurements using the X-ray photoelectron spectroscopy (XPS) of LaF_3_ and CeF_3_ were performed by the Scienta ESCA-300 spectrometer. The electronic gun was used to avoid charging the crystal surface.

The measurement of the luminescence parameters of crystals was performed at the SUPERLUMI station (DESY, HASYLAB) using synchrotron radiation [56]. The luminescence excitation spectra within the range of 4–20 eV, the luminescence spectra at the slit width of 0.5 nm in the region of 200–800 nm and the time constants of the luminescence decay with a time resolution of 0.2 ns were measured at 10 K.

## 3. Results and Discussions

**Figure 1:**
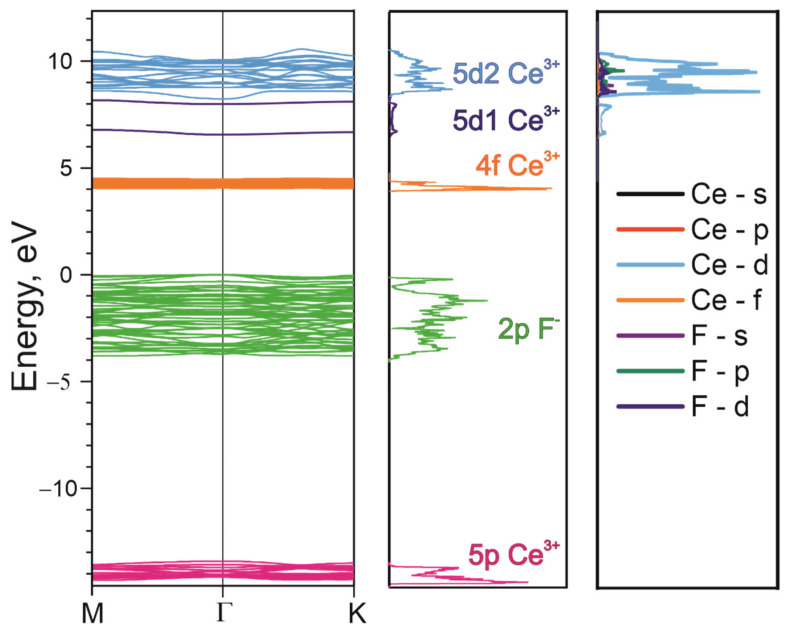
A fragment of ab initio electronic structure calculations of CeF_3_ by the PAW method. The total density of electronic states is given in the center. The partial density of electronic states is given on the right-hand side.

**Figure 2:**
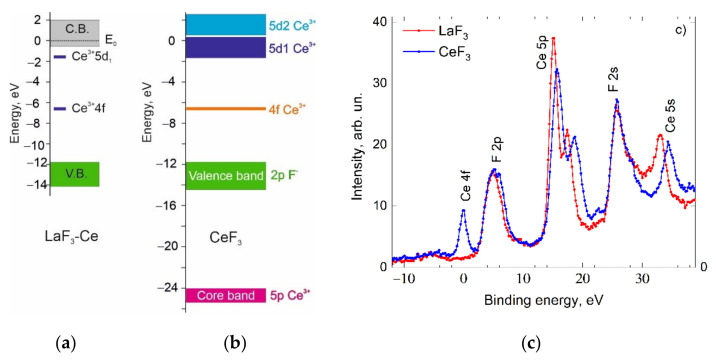
The location of energy levels in the cerium ion of LaF_3_ [57] (**a**), the tentative energy scheme for CeF_3_ (**b**) and the X-ray photoelectron spectra for LaF_3_ and CeF_3_ (**c**).

In order to achieve a certain understanding of the luminescence processes in CeF_3_, the calculation results of the energy band scheme by the PAW method and the hybrid exchange–correlational functional were analyzed. This method does not allow for the obtention of absolutely correct values for the energy gaps between different bands, but it provides information about the nature and electron density of states, the band order and reveals certain trends in the formation of the band structure. The fragment of the energy band scheme around the Г point and the electron density of states of the CeF_3_ crystal are presented in Figure 1. In this figure, we can distinguish the 5pCe^3+^ core band from the 2pF^−^ valence band. The narrow 4fCe^3+^ band is located at 4.5 eV above the top of the valence band, which is in good agreement with the XPS experimental data (Figure 2c). Figure 1 also shows the partial densities of the states for the region of excited cerium levels. As can be seen from Figure 1, the 5d states of Ce^3+^ are dominant in this area. The contributions of other states in this energy region are very small—at the level of a calculation error. Thereby, the conduction band is formed mainly by the 5d states of cerium. The value of the energy gap between the 4f and 5d states is underestimated and is equal to 3 eV, which is less than the experimental value (5 eV). Consider the features of the conduction band formation. The smaller dispersion of the 5d1-states (Figure 1) at the bottom of the conduction band at the Г point, compared with the 5d2 upper band states, indicates a larger effective electron mass in the 5d1-subband of the conduction band. The separation of the conduction band into two subbands (5d1 and 5d2) with significantly different effective masses was also observed in CeCl_3_ and CeBr_3_ crystals [48]. Using the formula to calculate the effective mass

(2) $$m*=\hbar {\left(\frac{\partial^{2}E}{\partial k^{2}}\right)}^{-1}$$

and the quadratic dispersion law around the Г point, we obtained the following values for the effective masses for the 5d1- and 5d2-subbands: m*_d1_ = 4.9 m_e_ and m*_d2_ = 0.9 m_e_. This feature allows us to consider the transitions between the 4f and 5d1 states as local transitions within the cerium ion (intracenter transitions), which leads to the formation of Frenkel excitons. The large effective mass of charge carriers for the 5d1-subband facilitates the localization of electrons with the formation of Frenkel self-trapped excitons as a result of the phonon relaxation of the exciton electronic component.

Since the first-principle band calculations give the underestimated energy parameters compared with the real values in the crystal, we have constructed a tentative energy band scheme (Figure 2b) based on the results of theoretical calculations and experimental data. To construct the energy scheme more accurately, we used experimental results: the data from the X-ray photoelectron spectra (Figure 2c), an empirical scheme of the energy position of cerium states in LaF_3_ by Dorenbos [57] (Figure 2a) and the energy parameters of the CeF_3_ luminescence excitation spectra. The X-ray photoelectron spectra indicate a similar position of 2pF- valence bands for CeF_3_ and LaF_3_ crystals. This allows for the placement of 2pF- levels in LaF_3_ and CeF_3_ at the same energy in the energy scale with respect to the vacuum. The position of the 4f-levels is taken from the XPS data (Figure 2c) and Dorenbos [57]. In Figure 2b, the structure of the conduction band is constructed according to the calculation results of the CeF_3_ energy band. Here, we emphasize the peculiarities of the conduction band, especially the existence of two separated 5d1 and 5d2 subbands with different effective masses. The presence of different subbands of the conduction band indicates the possibility of two types of optical transitions: (i) analogues of intracenter 4f-5d1 transitions with the subsequent localization of electronic excitations and (ii) 4f-5d2 transitions with cerium ionization.

**Figure 3:**
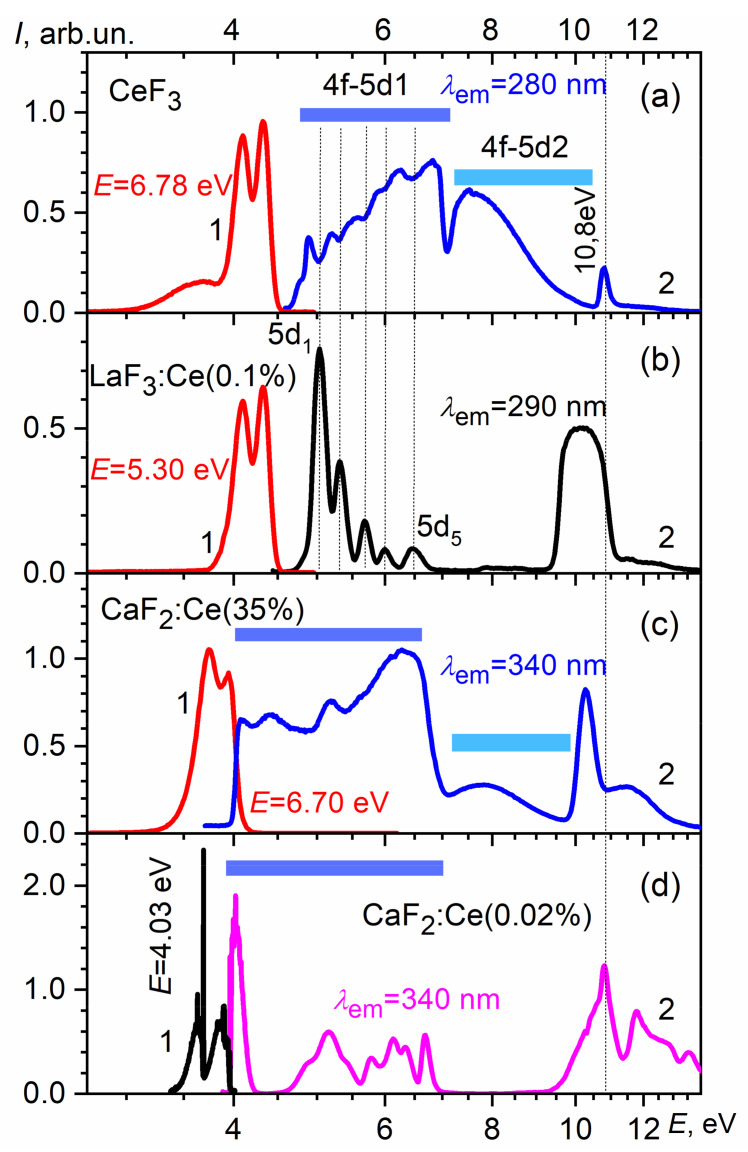
Luminescence (curves 1) and luminescence excitation spectra (curves 2) for: (**a**) 4f-5d cerium excitons in CeF_3_; (**b**) Ce^3+^ ions in LaF_3_:Ce; (**c**) CeF_3_ phases in CaF_2_:Ce (35 wt.%); (**d**) Ce^3+^ ions in CaF_2_: Ce (0.02 wt.%). T = 10 K.

Within the framework of the proposed energy scheme, certain luminescent features of the CeF_3_ crystals can be explained. The transitions between the 4f state and the localized 5d1 states cause the appearance of Frenkel excitons with the subsequent luminescence of regular Ce^3+^ ions at 282 and 305 nm (Figure 3a). The excitation spectra in the 4–6 eV range have a structure due to the splitting of the 5d levels by a low-symmetric crystal field (Figure 3a). Dips in the CeF_3_ spectra at 5.01, 5.30, 5.66, 5.97 and 6.45 eV correlate with the maxima of 5d_1_, …, 5d_5_ in the excitation spectra of the cerium luminescence in LaF_3_:Ce conditioned to 4f-5d transitions (Figure 3b). The presence of dips in the excitation spectra of the CeF_3_ luminescence at 280 nm is caused by non-radiative losses due to surface defects. The aforementioned similarity between the luminescence excitation spectra of CeF_3_ and LaF_3_:Ce was analyzed earlier in [58]. At 7.1 eV, a dip is observed in the excitation spectrum of CeF_3_, which may be caused by the presence of an energy gap between the 5d1 and 5d2 subbands of the conduction band. Taking into account the position of the 5d_1_ peak of the luminescence excitation band at 5.01 eV and the position of the dip at 7.1 eV, the distance between the bottom of the 5d1 and 5d2 subbands will be 2.1 eV. Using the method of induced absorption measuring in CeF_3_, the authors in [59] have obtained the energy of ~3 eV for the transition from the relaxed 5d1 state to the conduction band state (5d2). From these data, taking into account the magnitude of the Stokes shift (∆S = 1 eV) and its uniform distribution between the ground and excited states, we can determine that the distance between the unrelaxed 5d1 state and the 5d2 subband is equal to 2.5 eV, which is close to the 2.1 eV value found from the luminescence excitation spectra. The further structure of the excitation spectrum in the region of 7–10 eV within the framework of the proposed energy scheme may correspond to the transitions from 4f to the delocalized 5d2 states of the conduction band, accompanied by a cerium ionization (Ce^3+^ − e^−^ = Ce^4+^).

The nature of the peak at 10.8 eV (Figure 3a) may be due to the formation of the exciton states corresponding to the 2pF^−^→5d2 transitions. Exciton luminescence associated with the appearance of an anionic exciton is absent, possibly due to the deactivation of hole 2pF^0^-states by the electronic transitions from 4fCe^3+^ [60].

The evolution of the formation of the 5d2-conduction band can be understood by analyzing the luminescence excitation spectra of CaF_2_:Ce depending on the concentration of cerium ions [61]. For the CaF_2_:Ce (0.02 wt.%) sample in the region of 4–7 eV, there are bands corresponding to the intracenter 4f-5d transitions in the cerium ion. In the 7–10 eV region, the luminescence of cerium ions is not excited. Another situation is inherent for the CaF_2_:Ce (35 wt.%) sample. In the 7–10 eV region, an unstructured luminescence excitation band appears, which is characteristic for transitions from the 4f to 5d2 states of the conduction band in the CeF_3_ crystal. The appearance of such a structure in the luminescence excitation spectrum may indicate the formation of the CeF_3_ phase in the CaF_2_:Ce crystal (35 wt.%).

**Figure 4:**
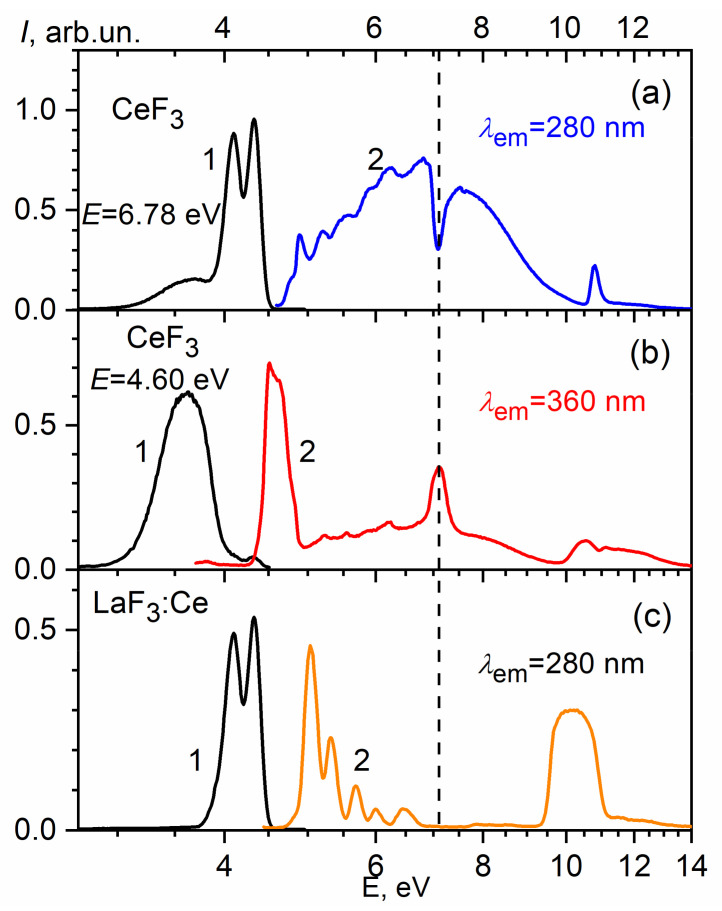
Luminescence (1) and luminescence excitation spectra (2) for: (**a**) cerium excitons in CeF_3_; (**b**) perturbed Ce centers in CeF_3_ crystals; (**c**) Ce^3+^-ions in LaF_3_:Ce. In all cases, T = 10 K.

**Figure 5:**
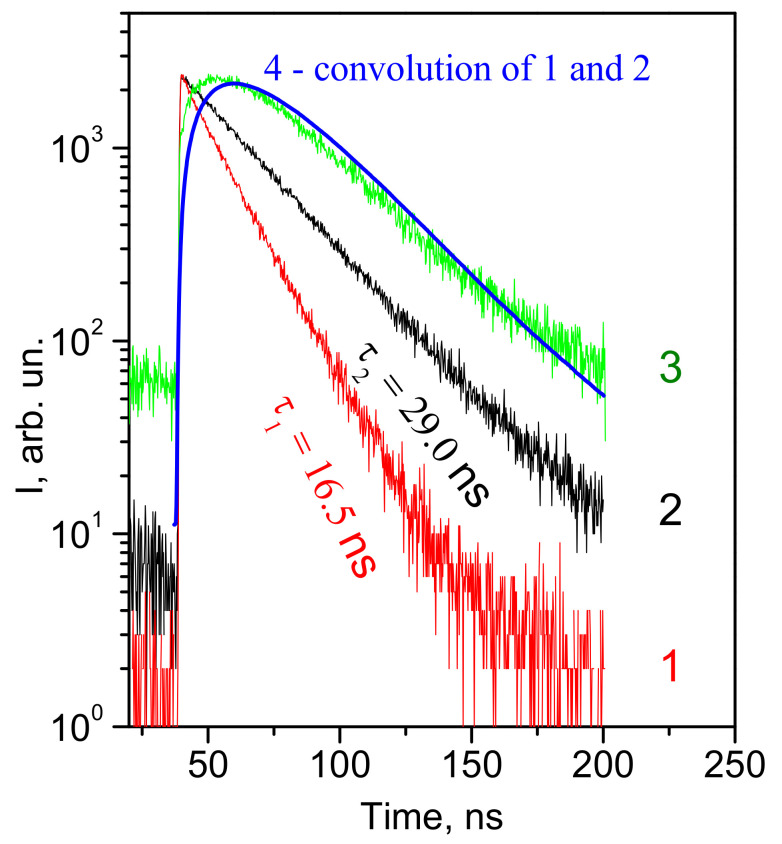
Kinetics of luminescence decay of 4f-5d cerium excitons (λ_em_ = 290 nm) for the excitation at λ_exc_ = 220 nm (1), luminescence band at λ_em_ = 340 nm for the excitation at λ_exc_ = 270 nm (2) and λ_exc_ = 220 nm (3). Convolution (curve 4) of 1 and 2 curves. T = 10 K.

Additional information about the energy structure features of the CeF_3_ crystal conduction band can be obtained by analyzing the excitation spectra of the luminescence band at 340 nm (Figure 4b). The peculiarity of this luminescence is the presence of excitation bands in the transparency region of the CeF_3_ host. The luminescence excitation maxima are located at the absorption edge of the host at 4 eV and at 7.1 eV (Figure 4b). The last one is located in the region of the relative transparency of the host, where the exciting light falls in the energy gap between the 5d2- and 5d1-subbands of the conduction band. Therefore, the luminescence at 340 nm is excited mainly outside the region of fundamental absorption of the host in the energy range where regular cerium ions do not absorb. The intensity of this luminescence in the absorption region of 4f-5d1 transitions is much lower, and the luminescence, in this case, is rather due to the reabsorption of the 4f-5d exciton emission. The analysis of luminescence decay curves can approve it. Figure 4 and Figure 5 shows the luminescence decay curve of 4f-5d excitons (curve 1), the luminescence decay curve for the band at 340 nm for excitation in the region of host transparency (270 nm) (2) and in the region of 4f-5d exciton creation (λ_exc_ = 220 nm) (curve 3). The convolution (curve 4) of the exciton luminescence decay curve (curve 1) and the luminescence at 340 nm excited in the transparency region (curve 2) coincide with the decay kinetics of the band peaked at 340 nm excited in the host absorption region (curve 3). This coincidence confirms the radiative mechanism of the energy transfer from 4f-5d excitons to the radiation centers responsible for the luminescence at 340 nm. A similar conclusion about the mechanism of energy transfer has been made in [62].

## 4. Conclusions

First-principle calculations of CeF_3_ crystals by the PAW method using the hybrid exchange–correlational functional PBE0 reveal peculiarities of the conduction band structure, particularly the existence of energetically separated subbands with different effective electron masses m*_d1_ = 4.9 m_e_ (5d1 subband) and m*_d2_ = 0.9 m_e_ (5d2 subband). Such values of effective masses assume the availability of localized electron states of the 5d1 subband and delocalized states of the 5d2 subband. From this point of view, the 4f-5d1 transitions may correspond to the intracenter transitions in the Ce^3+^ ion, which facilitates the appearance of Frenkel excitons, and the 4f-5d2 transitions may be associated with the ionization of cerium ions. The energy gap between the 5d2 and 5d1 subbands appears as a dip at 7.1 eV in the exciton luminescence excitation spectra or as a maximum at 7.1 eV in the excitation spectra of the luminescence band peaked at 340 nm. The mechanism of energy transfer from the Frenkel cerium excitons to the luminescence centers responsible for the band at 340 nm is radiative. The anionic exciton corresponding to the 2pF-5d2 transition is responsible for the band peaked at 10.8 eV in the excitation spectrum of cerium luminescence.

## Data Availability

Data sharing is not applicable to this article.
